# A torque-based method demonstrates increased rigidity in Parkinson’s disease during low-frequency stimulation

**DOI:** 10.1007/s00221-012-3107-7

**Published:** 2012-05-13

**Authors:** Simon Little, Raed A. Joundi, Huiling Tan, Alek Pogosyan, Beth Forrow, Carole Joint, Alexander L. Green, Tipu Z. Aziz, Peter Brown

**Affiliations:** 1 Department of Clinical Neurology, 6th Floor, West wing, John Radcliffe Hospital, Oxford University, Headley Way, Oxford, OX3 9DU UK; 2 Sobell Department of Motor Neuroscience and Movement Disorders, UCL Institute of Neurology, Queen Square House, Queen Square, London, WC1N 3BG UK

**Keywords:** Parkinson’s disease, Rigidity, Oscillations, Beta, Deep brain stimulation

## Abstract

Low-frequency oscillations in the basal ganglia are prominent in patients with Parkinson’s disease off medication. Correlative and more recent interventional studies potentially implicate these rhythms in the pathophysiology of Parkinson’s disease. However, effect sizes have generally been small and limited to bradykinesia. In this study, we investigate whether these effects extend to rigidity and are maintained in the on-medication state. We studied 24 sides in 12 patients on levodopa during bilateral stimulation of the STN at 5, 10, 20, 50, 130 Hz and in the off-stimulation state. Passive rigidity at the wrist was assessed clinically and with a torque-based mechanical device. Low-frequency stimulation at ≤20 Hz increased rigidity by 24 % overall (*p* = 0.035), whereas high-frequency stimulation (130 Hz) reduced rigidity by 18 % (*p* = 0.033). The effects of low-frequency stimulation (5, 10 and 20 Hz) were well correlated with each other for both flexion and extension (*r* = 0.725 ± SEM 0.016 and 0.568 ± 0.009, respectively). Clinical assessments were unable to show an effect of low-frequency stimulation but did show a significant effect at 130 Hz (*p* = 0.002). This study provides evidence consistent with a mechanistic link between oscillatory activity at low frequency and Parkinsonian rigidity and, in addition, validates a new method for rigidity quantification at the wrist.

## Introduction

Recordings of local field potentials (LFPs) from the basal ganglia of patients with Parkinson’s disease have shown prominent oscillatory activity at frequencies under about 30 Hz (Alonso-Frech et al. 2006; Bronte-Stewart et al. 2009; Brown et al. 2001; Cassidy et al. 2002; Foffani et al. 2005; Kühn et al. 2006; Marceglia et al. 2006; Weinberger et al. 2006). Such activity is suppressed by treatment with levodopa and by high-frequency deep brain stimulation (Jenkinson and Brown 2011). Oscillatory activity has been shown to correlate with rigidity-bradykinesia both in the rest state and in response to treatment, although the frequency band of interest has varied between studies from 8 Hz up to 35 Hz (Brown and Williams 2005; Chen et al. 2010; Kühn et al. 2006; López-Azcárate et al. 2010; Pogosyan et al. 2010; Ray et al. 2008; Zaidel et al. 2010). Indeed, to date, there is little evidence that any particular low frequency of oscillation is any more predictive of motor deficit than another (Kühn et al. 2009). The above correlative evidence of a link between oscillatory activity across a fairly wide range of low frequencies and bradykinesia has been strengthened by a number of interventional studies in which bradykinesia has been exacerbated by stimulation of the subthalamic nucleus (STN) at 5, 10 and 20 Hz although effect sizes have been small (Chen et al. 2007, 2011; Eusebio et al. 2008; Fogelson et al. 2005; Timmermann et al. 2004).

Hitherto, however, there has been no evidence to support a direct causal link between low-frequency oscillatory activity and rigidity. Here, we test for such a link by stimulating the STN at low frequencies in patients with Parkinson’s disease while we assess rigidity with an objective mechanical device that allows continuous scalar estimates of tone. We examined patients on their usual antiparkinsonian medication, so as to avoid potential ceiling effects whereby rigidity could not worsen further with low-frequency stimulation. We hypothesized that stimulation at frequencies ≤20 Hz would increase rigidity at the wrist, while stimulation at clinically effective high frequencies would reduce rigidity.

## Methods

### Subjects and surgery

The study was approved by the local ethics committee and subjects gave their informed, written consent. Twelve patients (mean age, 61.5 ± SEM 1.9 years; disease duration, 13.1 ± 1.6 years; see Table 1 for further details) with idiopathic Parkinson’s disease were investigated 2.9 ± 0.8 years after implantation of bilateral deep brain stimulation (DBS) electrodes into the STN. Indications for surgery were advanced Parkinsonism with motor fluctuations and/or dyskinesias or tremor that could not be sufficiently controlled by drugs. The DBS electrode used was model 3389 (Medtronic Neurological Division, Minneapolis, USA) with four platinum–iridium cylindrical surfaces (1.27-mm diameter and 1.5 mm length) and centre-to-centre separations of 2 mm. Contacts 0 and 3 were the most caudal and rostral contacts, respectively. STN electrode trajectories were aimed at the dorsolateral STN. The STN was identified on high-resolution T2 weighted axial, magnetic resonance images. On the day of surgery, a Cosman-Roberts-Wells^®^ (Radionics, Burlington, MA) stereotactic base ring was applied to the patient’s scalp under local anaesthetic. A stereotactic computed tomography (CT) scan was obtained, and the images were fused to the MR using Radionics Stereoplan^®^ software. The STN was identified and targeted visually although concordance with the Schaltenbrand and Wahren atlas (1977) was confirmed. The electrodes were introduced via a 2.7-mm twist drill craniotomy in all cases. Correct placement of DBS electrodes in the STN was supported intraoperatively by loss of rigidity and/or tremor suppression with stimulation and postoperatively by performing a stereotactic CT scan that was fused to the T2-weighted MR images as above. Operations were performed in two stages with implantation of the pulse generator after 1 week of testing to confirm clinical effect.

**Table 1:** Clinical details of patients

Case	Age (years)	Disease duration/time since operation (years)	Predominant symptom	Preop levodopa challenge UPDRS part III on/off^c^	Rigidity (L + R arm) off/on HFS^a^	Medication (total daily dose)	Chronic stimulation parameters
1	65	24/2	Tremor (R side)	14/32	1.5/0	Co-beneldopa 1,125 mg Ropinirole 8 mg Amantadine 100 mg	L 3.1 V 60 μs 130 Hz R 2.6 V 60 μs 130 Hz
2	56	6/1	Tremor (R side)	22/43	5/2.5	Co-careldopa 1,000 mg Entacapone 800 mg Selegeline 10 mg Co-beneldopa 125 mg	L 3.9 V 60 μs 130 Hz R 1.5v 60 μs 130 Hz
3	60	11/6	R sided rigidity/tremor	28/45	3/2.5	Co-careldopa 312.5 mg	L 3.4 V 60 μs 180 Hz R 2.9 V 60 μs 180 Hz
4	71	17/2	Dyskinesias	8/38	3/1	Co-beneldopa 1,725 mg Amantadine 200 mg	L 2.8 V 60 μs 130 Hz R 3.3 V 60 μs 130 Hz
5	65	18/2	Rigidity L side	7/34	1.5/2	Co-beneldopa 1,000 mg	L 2.8 V 60 μs 130 Hz R 3.2 V 60 μs 130 Hz
6	68	10/0.5	Bradykinesia	2/17	0.5/1	Rotigitine 4 mg Co-beneldopa 1,000 mg	L 3.4 V 60 μs 130 Hz R 3.4 V 90 μs 130 Hz
7	49	10/2	R leg dyskinesia. Bradykinesia/tremor	10/35	2/0.5	Co-careldopa 500 mg Pramipexole 2.1 mg	L 2.6 V 90 μs 130 Hz R 2.3 V 60 μs 130 Hz
8	64	12/2	R sided rigidity	17/47	3/3	Co-beneldopa 500 mg Tolcapone 300 mg	L 3.6 V 90 μs 130 Hz R 3.2 V 90 μs 130 Hz
9	62	13/3	R sided tremor and dyskinesias	15/38	3.5/2	Co-beneldopa 1,125 mg Ropinirole 24 mg	L 3.9 V 90 μs 130 Hz R 2.4 V 60 μs 130 Hz
10	56	19/10	Bradykinesia/rigidity	^b^/51 (off)	4/4	Co-careldopa 375 mg Ropinirole 8 mg	L 3.5 V 90 μs 185 Hz R 3.1 V 120 μs 185 Hz
11	55	6/1.5	Leg tremors. Drug induced nausea	6/27	7.5/3.5	Co-careldopa 500 mg	L 3.5 V 90 μs 130 Hz R 3.4 V 20 μs 130 Hz
12	67	11/2.5	Severe off periods	7/23	2.5/0.5	Co-beneldopa 312.5 mg Ropinirole 3 mg	L 3 V 60us 130 Hz R 3.1 V 60 μs 130 Hz

^a^Assessed as item 22 of motor section of UPDRS

^b^Preop off drugs score missing

^c^UPDRS III tested after overnight withdrawal of all antiparkinsonian medication and again after a test dose of a minimum of 200 mg levodopa. Test performed less than 3 months before surgery

### Protocol

All patients were assessed on their usual medication. Experiments were timed to begin at the mid-point of the drug dosing at least 1 h after last dose. Clinical ‘on’ state was confirmed both by the patient and also by the attending neurologist. Patients were studied with the STN stimulation switched off and during bilateral STN stimulation at 5, 10, 20, 50 Hz and their usual therapeutic high-frequency setting. The latter will be termed 130 Hz, although in two patients, therapeutic stimulation was delivered at a higher frequency (see Table 1). The order of stimulation frequencies (including no stimulation) was pseudo-randomized across patients, with the exception that, to minimize protocol length, the final block was always the therapeutic high-frequency setting. Stimulation contacts, amplitude and pulse duration remained the same as utilized for chronic therapeutic stimulation in each patient (see Table 1). The study was performed in a double-blind manner with both the patient and assessor of rigidity unaware of which stimulation frequency was being used between 0 and 50 Hz. Eight minutes elapsed after changing stimulation settings before testing rigidity.

### Rigidity assessment

Rigidity at each wrist was clinically assessed with item 22 of the motor section of the Unified Parkinson’s disease rating scale (UPDRS). Half-points were used to increase sensitivity (Chen et al. 2010; Kühn et al. 2006). Thus, rigidity scores were defined as 0—Absent; 0.5—Slight or detectable when activated by mirror or other movements but present only occasionally/intermittently; 1—Consistently slight or detectable only when activated by mirror or other movements; 1.5—Rigidity detectable consistently but very mild; 2—Mild to moderate; 2.5—Moderate to marked; 3—Marked, but full range of motion easily achieved; 3.5—Marked with some mild difficulty in achieving full range of motion; 4—Severe, range of motion achieved with difficulty; 4.5—Very severe, full range of motion limited. However, clinical assessment alone has poor sensitivity as well as high inter-rater and intra-rater variability (Patrick et al. 2001; Prochazka et al. 1997). We, therefore, followed clinical assessment with an objective mechanical assessment of rigidity at each wrist that afforded continuous scalar estimates. This was preferred to an index of rigidity derived from EMG, as several studies have confirmed that torque-based methods of quantification are more strongly related to clinically determined rigidity than electromyography-based metrics (Endo et al. 2009; Levin et al. 2009; Park et al. 2011).

**Fig. 1:**
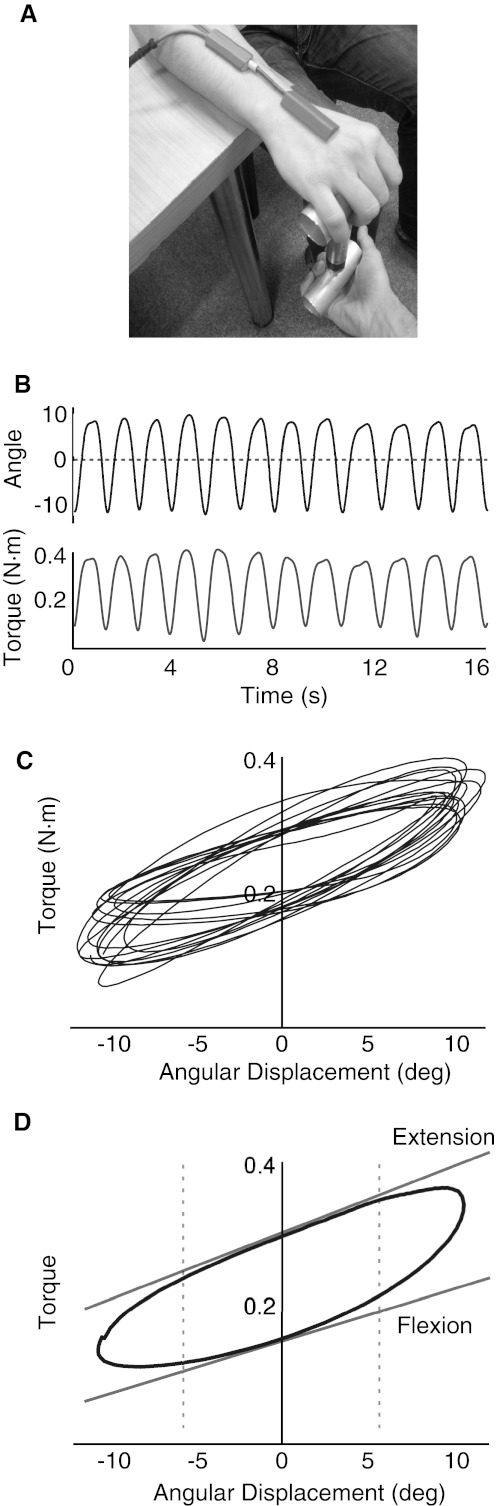
**a** Photograph of mechanical rigidity device. **b** Time series of force and displacement during stimulation at 5 Hz from subject 7 (UPDRS—clinical rigidity score—1). **c** Superimposed torque–angular displacement cycles during stimulation at 5 Hz. **d** Schematic of analytical method for determining rigidity coefficients in flexion and extension. A single displacement cycle is shown from stimulation at 5 Hz (*blue line*) with linear regression of mid-cycle phase (*dashed lines* 50 % of cycle) for both flexion and extension (*red line*). The gradient of the regression line is taken as the rigidity for that cycle

We assessed wrist torque in response to externally based displacement imposed by the examiner rather than by a motor. The former was preferred so as to limit anxiety and reinforcement related to the use of a motor and fixed manipulandum. Angular displacement was measured using an electronic goniometer across the wrist (TMS International B.V., Netherlands) that was calibrated using a manual goniometer for each patient across the whole angular range of displacement. Force was measured using a strain gauge (Omegadyne LCM201-100N) mounted between two horizontal aluminium bars (Fig. 1a). The strain gauge had a linear range from 0 to 100 N ± 1 %. Force and angle measures were low-pass filtered at 1 kHz, sampled with a frequency of 2,048 Hz and recorded through a commercial amplifier (TMSI Port 7, TMS International B.V., The Netherlands).

The metacarpophalangeal junction of the subject’s hand was rested on the top bar of the device while the bottom bar was controlled by the experimenter. The distance between the MCP joint and the wrist was used to calculate torque (force x distance). Patients were requested to look straight ahead and minimize movement and speech. The hand was then passively flexed and extended as the experimenter applied force in a sinusoidal, vertical, manner. Velocity was controlled by delivering a fixed displacement at a set frequency of 0.75 Hz using a visual metronome that was visible to the experimenter but not the patient. This frequency falls in the range used clinically to determine rigidity with the motor UPDRS (Shapiro et al. 2007). The experimenter moved the wrist through half the full range of comfortable displacement around the horizontal plane. Patients were encouraged to fully relax and not to assist with movement. Active assistance was identified by subjective reduction in force on the strain gauge device prior to the start of a cycle, and these sections were removed and rigidity assessment repeated after patient re-instruction. A minimum of 14 cycles per condition was performed.

### Analysis

We derived the elastic coefficient of the wrist calculated from unit torque/unit angular displacement (Endo et al. 2009; Powell et al. 2012). Rigidity was calculated offline in MATLAB (v. 7.11.0, R2010b, The Mathworks, Natik, MA, USA) using custom written scripts. The oscillatory time series of both torque and angular displacement were pass-band filtered between 0.25 and 1.25 Hz using a 4th order Butterworth filter to remove offset, noise and any super-added tremor. Continuous oscillatory traces were broken into individual cycles using the phase determined through a Hilbert transform. Displacement was plotted against torque (*x* and *y*, respectively) for the central 50 % of each cycle, and this was fitted with a linear regression line (Fig. 1b). The gradient of this line was taken as the elastic coefficient and the procedure repeated for each cycle of movement. The mean elastic coefficient of the last 12 cycles performed in each experimental run was taken as our index of rigidity for that condition. Exclusion of the first few movement cycles allowed the subject to relax before assessments were made. This procedure was separately performed for both the flexion and extension phase of each cycle.

All rigidity values were normalized to the off-stimulation state [(stim off − stim (*f*)/stim off)] to determine percentage change compared to baseline, and signs inverted so that a positive % change represented an increase in rigidity. Kolmogorov–Smirnov tests confirmed the normality of the % changes estimated with the device, and so stimulation frequency effects were evaluated with *t* tests. Significant effects were reported if they remained significant according to the False Discovery Rate procedure and *t* tests were two tailed. Changes in clinical assessments of rigidity were assessed with non-parametric Wilcoxon signed rank tests. Clinical assessments of rigidity were logarithmically transformed prior to correlation with device measurements, given the known logarithmic nature of psychophysical observation (Weber’s law). Correlations were performed using Spearman’s correlation. Statistical analysis was performed in the Statistical Program for Social Sciences (SPSS) statistical software (version 17.0, SPSS Inc., Chicago, IL, USA).

## Results

**Fig. 2:**
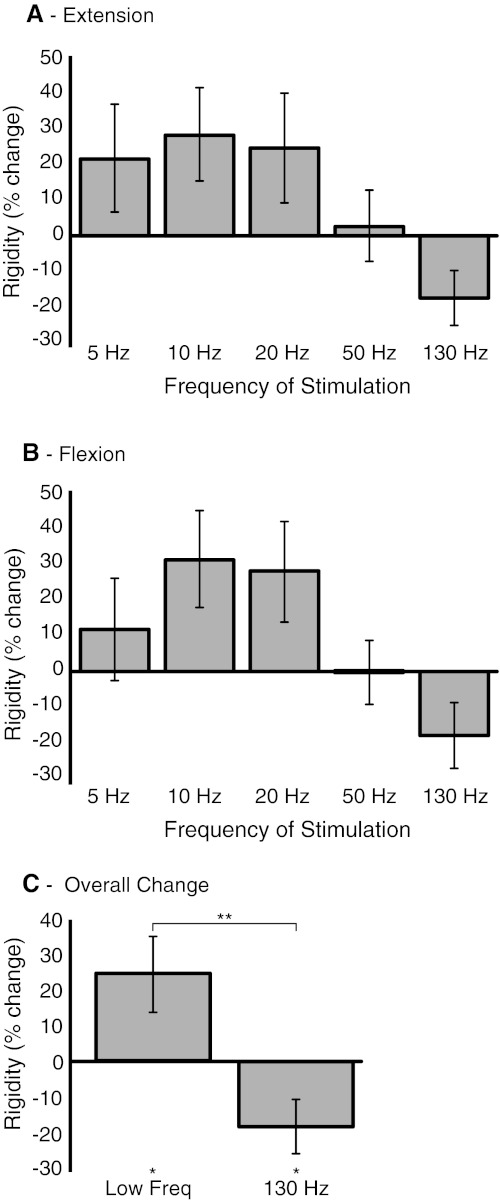
Effects of bilateral stimulation of the STN at different frequencies on quantitative rigidity compared to off-stimulation state. **a** Mean (±SEM) percentage change in extension coefficients. **b** Percentage change in flexion coefficients. Stimulation at frequencies ≤20 Hz exacerbates rigidity, whereas stimulation at the therapeutic frequency of 130 Hz tends to improve rigidity. The pattern is similar for extension and flexion. **c** Percentage change of averaged low-frequency (5,10 and 20) flexion/extension coefficients with significance. Low-frequency stimulation significantly increases rigidity (*p < 0.05), and high-frequency stimulation significantly reduces rigidity (*p < 0.05). Low-frequency and high-frequency stimulation are significantly different (**p < 0.01). Twenty-four upper limbs were tested with patients on Parkinsonian medication

Our a priori hypothesis was that the effects of stimulation at frequencies ≤20 Hz would be related and lead to an increase in rigidity, whereas the effect of stimulation at 130 Hz would lead to a decrease in rigidity at the wrist. Objective assessment of tone with our device bores this out (Fig. 2). The effects of stimulation at 5, 10 and 20 Hz all correlated with one another, whether extension or flexion was tested (mean Fisher transformed *r* = 0.725 ± SEM 0.016 and 0.568 ± 0.009, respectively, all correlations individually significant, *p* < 0.05). However, correlations between the effects of stimulation at 5–20 Hz with those of stimulation at 130 Hz were weak (*r* = 0.229 ± 0.042 and *r* = 0.308 ± 0.121 for extension and flexion, all but one non-significant). The similarity in the response to low-frequency (5, 10 and 20 Hz) stimulation in flexion and extension seen at the group level (Fig. 2) was also found at the level of individual limbs. Thus, the maximum rigidity in the low-frequency blocks in flexion was at the same stimulation frequency as in extension in 19 out of 24 sides (Fisher’s exact test, two-tailed *p* = 0.0032, compared to the 8 out of 24 instances expected by chance). Across movement phases, the maximum rigidity in the low-frequency blocks was at 5 Hz on 17 sides, 10 Hz on 17 sides and 20 Hz on 14 sides.

Thus, effects on flexion and extension were very similar (contrast Fig. 2a, b), and so in subsequent analyses, we averaged changes across flexion and extension. Thereafter, we averaged the effects of 5–20 Hz stimulation and compared this to baseline (no stimulation) and to the effect of stimulation at therapeutic frequency, 130 Hz. Low-frequency stimulation (5–20 Hz) increased rigidity by 24.0 % (one-sample *t* test, *t*(*df* 23) = 2.240, *p* = 0.035), whereas high-frequency stimulation reduced rigidity by −17.8 % (one-sample *t* test, *t*(*df* 23) = −2.284, *p* = 0.033). The effects of low- and high-frequency stimulation were also different (paired *t* test, *t*(*df* 23) = 3.511, *p* = 0.002).

**Fig. 3:**
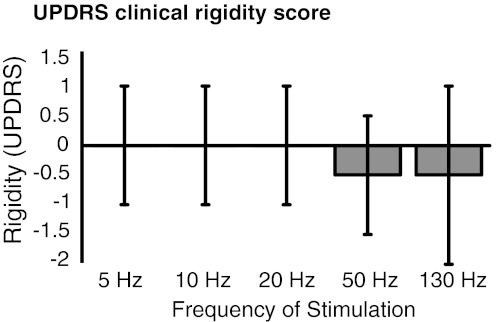
Effects of bilateral stimulation of the STN at different frequencies on clinical UPDRS rigidity scores compared to off-stimulation state. Median and inter-quartile ranges are shown. Rigidity was assessed using item 22 of the motor UPDRS. Twenty-four upper limbs were tested with patients on Parkinsonian medication

**Fig. 4:**
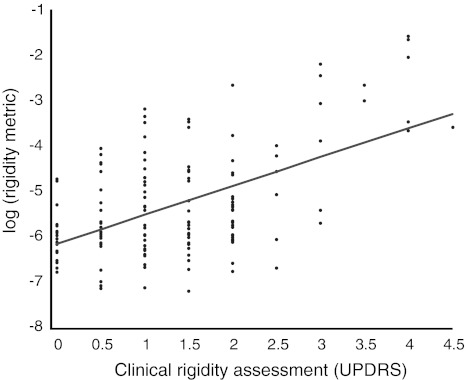
Scatter plot of clinical rigidity assessment versus log quantitative rigidity scores. There is a correlation between clinical rigidity scores and log rigidity scores, rho = 0.361, *p* = 0.002. Rigidity was clinically assessed using item 22 of the motor UPDRS. Data are compiled from 24 upper limbs and five different stimulation frequencies (including 0 Hz). Patients were on Parkinsonian medication

We were unable to demonstrate an effect of low-frequency stimulation when using double-blinded clinical assessment of rigidity (Fig. 3). As above, we averaged the effects of 5–20 Hz stimulation and compared this to baseline and to the effect of stimulation at therapeutic frequency, 130 Hz. Low-frequency stimulation increased rigidity by 1 % (Wilcoxon test, *p* = 0.87), whereas high-frequency stimulation reduced rigidity by 45 % (Wilcoxon test, *p* = 0.002). The effects of low- and high-frequency stimulation were also different (Wilcoxon test, *p* = 0.002). Clinical and device assessments were significantly correlated, although the relationship was not strong (Spearman’s rho = 0.382, *p* = 0.002; Fig. 4).

## Discussion

This study shows a worsening of objectively recorded rigidity in patients with Parkinson’s disease during low-frequency stimulation of the subthalamic nucleus and thus provides further evidence consistent with a causal role for these oscillations in the pathophysiology of the condition. Recordings of the effects of low-frequency stimulation were made under double-blind conditions. Clinical assessment of tone made under the same conditions, however, did not reveal any change, perhaps because of the acknowledged poor sensitivity and high variability of this technique (Patrick et al. 2001; Prochazka et al. 1997). The latter may relate to its subjectivity and its rating on a bounded, ordinal rating scale. The poor sensitivity and high variability of clinical assessment may also explain the relatively modest correlation between mechanical and clinical assessments of rigidity in the present investigation. Such modest but significant correlations between objective and clinical assessments of rigidity have also previously been reported in the setting of DBS (Levin et al. 2009).

Previous studies have investigated the effect of low-frequency stimulation on bradykinesia in patients with Parkinson’s disease and generally found a modest, deleterious effect with stimulation over 5–20 Hz raising doubt on the scope of the mechanistic role of low-frequency oscillations (Chen et al. 2011). The results presented here are remarkable for a larger effect size (24 %) and for extending evidence of a link between low-frequency stimulation and impairment to rigidity. As previous studies have all been performed off medication, it is possible that a floor effect limited the size of previous results, though it is also possible that rigidity is more sensitive than bradykinesia to low-frequency stimulation. In studying patients on medication, we limited any confounding floor effect; however, it should be acknowledged that baseline rigidity may be more variable on medication than off medication, and this may have contributed to the variance in our recordings.

Still, although the current results provide evidence consistent with a mechanistic link between oscillatory activity at low frequency (in this case driven by external stimulation), and rigidity, our effect size was still only moderate. As discussed elsewhere, this may have arisen because of the imperfect nature of our intervention with respect to temporal and spatial patterning, so that electrical stimulation was not a precise mimic of spontaneous oscillatory activity in the circuit (Brown 2007). Nevertheless, the precise scale of the contribution of low-frequency stimulation to rigidity in Parkinson’s disease remains unclear.

Both objective and clinical assessments confirmed that high-frequency stimulation improved rigidity in our series of patients on medication, although clinical assessments may have been biased by the fact that the assessment of stimulation at 130 Hz was performed last and was not double blinded. It is well established that high-frequency stimulation of the subthalamic nucleus improves rigidity in patients withdrawn from medication (Benabid et al. 2009), and some investigations, as here, have also reported improvements during stimulation in medicated patients (Maurer et al. 2003; Raoul et al. 2012).

Our data reinforce previous studies showing a correlation between low-frequency synchrony and aggregate measures of bradykinesia and rigidity (Brown and Williams 2005; Chen et al. 2010; Kühn et al. 2006; López-Azcárate et al. 2010; Pogosyan et al. 2010; Ray et al. 2008) and of rigidity alone (Hammond et al. 2007; Zaidel et al. 2010). One of the latter studies reported peak correlations with rigidity at 15 Hz (Zaidel et al. 2010). With respect to rigidity, it is possible that low-frequency synchrony in basal ganglia-cortical loops may, under physiological conditions, promote postural activity through the upregulation of the effects of sensory inputs that reinforce such activity (Androulidakis et al. 2006; Gilbertson et al. 2005; Lalo et al. 2007). This upregulation may be further heightened when low-frequency synchrony is exaggerated in Parkinson’s disease (Hammond et al. 2007). This, however, remains speculative.

Finally, the present study introduces a new method of rigidity assessment in patients with Parkinson’s disease which, although simple to implement, mimicking clinical evaluation, affords objective, continuous scalar estimates of tone, rather than the bounded, ordinal clinical assessment using the motor UPDRS.
